# Injection Drug Use Is a Risk Factor for HCV Infection in Urban Egypt

**DOI:** 10.1371/journal.pone.0007193

**Published:** 2009-09-28

**Authors:** Adela Paez Jimenez, Mostafa K. Mohamed, Noha Sharaf Eldin, Hasnaa Abou Seif, Said El Aidi, Yehia Sultan, Nasr Elsaid, Claire Rekacewicz, Mostafa El-Hoseiny, May El-Daly, Mohamed Abdel-Hamid, Arnaud Fontanet

**Affiliations:** 1 Emerging Disease Epidemiology Unit, Institut Pasteur, Paris, France; 2 Department of Community, Environmental and Occupational Medicine, Faculty of Medicine, Ain Shams University, Cairo, Egypt; 3 Imbaba Fever Hospital, Cairo, Egypt; 4 Abassaia Fever Hospital, Cairo, Egypt; 5 Ministry of Health and Population, Cairo, Egypt; 6 Viral Hepatitis Research Laboratory, National Hepatology & Tropical Medicine Research Institute, Cairo, Egypt; 7 National Liver Institute, Menoufia University, Menophya, Egypt; 8 Department of Microbiology, Faculty of Medicine, Minia University, Minia, Egypt; University of Liverpool, United Kingdom

## Abstract

**Objective:**

To identify current risk factors for hepatitis C virus (HCV) transmission in Greater Cairo.

**Design and Setting:**

A 1∶1 matched case-control study was conducted comparing incident acute symptomatic hepatitis C patients in two “fever” hospitals of Greater Cairo with two control groups: household members of the cases and acute hepatitis A patients diagnosed at the same hospitals. Controls were matched on the same age and sex to cases and were all anti-HCV antibody negative. Iatrogenic, community and household exposures to HCV in the one to six months before symptoms onset for cases, and date of interview for controls, were exhaustively assessed.

**Results:**

From 2002 to 2007, 94 definite acute symptomatic HCV cases and 188 controls were enrolled in the study. In multivariate analysis, intravenous injections (OR = 5.0; 95% CI = 1.2–20.2), medical stitches (OR = 4.2; 95% CI = 1.6–11.3), injection drug use (IDU) (OR = 7.9; 95% CI = 1.4–43.5), recent marriage (OR = 3.3; 95% CI = 1.1–9.9) and illiteracy (OR = 3.9; 95% CI = 1.8–8.5) were independently associated with an increased HCV risk.

**Conclusion:**

In urban Cairo, invasive health care procedures remain a source of HCV transmission and IDU is an emerging risk factor. Strict application of standard precautions during health care is a priority. Implementation of comprehensive infection prevention programs for IDU should be considered.

## Introduction

The highest HCV prevalence in the world occurs in Egypt at an estimated 12% [1], i.e. 10 to 20 fold higher than in Northern Europe [2] or in the United States [3]. The bulk of chronic infection is age-related [4] and occurs among persons of rural origin. Cohort studies have estimated a 9% prevalence and 0.8/1000 person-years incidence in Upper Egypt, and a 24% prevalence and 6.8/1000 incidence in the Nile Delta [5], [6].

The widespread schistosomiasis treatment campaigns with intravenous tartar emetic, carried out in the countryside in the 60's- early 80's, ignited this epidemic through reuse of insufficiently sterilised needles and syringes [7]. Since then, cross-sectional studies have shown unsafe injection practices, history of blood transfusion, invasive medical procedures, and instrument-assisted birth deliveries as associated with HCV infection [8]–[10]. Intra-familial transmission may also have played an important role, as evidenced in two recent cohort studies [6], [11].

However, HCV transmission has been studied almost exclusively in rural areas, with only two uncontrolled studies reporting on urban hepatitis C patients [12], [13]. After last decades of large rural exodus leading to the suburbs of Cairo, 45% of the Egyptian population is urban (Source CAPMAS, 2000). In order to identify current risk factors for HCV infection in urban Egypt, we have conducted a case-control study recruiting incident HCV case patients, i.e. recently acquired infections, in two hospitals serving Greater Cairo (Cairo and its suburbs).

## Methods

### Participants' recruitment and questionnaire

From April 2002 to June 2007, a 1∶1 matched case-control study with two control groups was conducted. Incident acute symptomatic hepatitis C patients were enrolled as cases either (i) before seroconversion, with negative anti-HCV antibody and positive HCV RNA laboratory results or (ii) with rapid seroconversion: positive anti-HCV antibody and positive HCV RNA associated with alanine aminotransferase (ALT) ≥20 times the ULN (≥800 IU/L). The high ALT threshold was used to exclude ALT flares in patients with chronic hepatitis C.

Two control groups were matched on age (±1 year) and sex: household members (family controls) and acute hepatitis A patients (HAV controls) diagnosed at the same hospitals. Only laboratory confirmed anti-HCV negative controls were included in the study.

Since age at infection profile of HAV and HCV are overlapping in the age group between 15–40 years of age, only cases and controls in this age range were considered to allow proper matching.

Acute hepatitis patients were identified at the two ‘fever’ hospitals of Greater Cairo, Abbassia and Imbaba. ‘Fever’ Hospitals are large public and non-paying hospitals whose patient population derives mostly from low socioeconomic groups (methods published elsewhere [12]). In- and outpatients with recent (<3 weeks) symptoms suggestive of hepatitis (fever or jaundice) were invited to participate in the study. After providing written informed consent (from one of the parents if less than 18 years of age), they answered orally administered standardised questionnaires covering socio-demographic characteristics, present and past health conditions, and exposure to potential risk factors for viral hepatitis in the one to six months before onset of illness. Regarding socio-demographic variables, illiteracy (the inability to read and write) and marriage duration (aggregated on recently married versus married for longer than one year) were of special interest.

Other exposures in the one to six months before symptoms onset included a history of invasive medical procedures (e.g., surgery, intravenous catheter, endoscope, blood transfusion, IV drip infusions, injections, haemodialysis, biopsy, endoscope, sclerotherapy of varicose veins), obstetrical procedures (caesarean section, episiotomy, uterine curettage) and dental treatment. Information on the following community–acquired exposures was also collected: circumcision, ear-piercing, shaving at barbershops, sharing razors or nail trimmers with family members, manicure or pedicure at beauty salons, tattooing, and acupuncture. Due to their sensitive nature, questions on high-risk behavior such as alcohol consumption, drug use (e.g., sniffing or intravenous (IDU)) and multiple sexual partners were only asked to men. During counselling, case patients were invited to ask their families to participate in the study. After providing informed consent, family members answered the same questionnaire regarding exposures at risk in the one to 6 months before interview.

Approval for the study was obtained from the Institutional Review Board of the Egyptian Ministry of Population and Health (MoPH) and the Ethics Committee of the National Hepatology and Tropical Medicine Research Institute (NHTMRI, Egypt).

### Laboratory testing

A 10 ml venous blood sample was collected. Patients were tested for standard liver functions (ALT), aspartate aminotransferase (AST), total and indirect bilirubin, alkaline phosphatase) and for the following hepatitis markers: anti-HAV IgM (HAVAB®, M EIA, Abbott Laboratories, Diagnostics Division, IL, USA), anti-HBc IgM (CORZYME®, M rDNA, Abbott Laboratories, Diagnostics Division, IL, USA) and HBs antigen (AUSZYME MONOCLONAL®, third generation EIA, Abbott Laboratories, Diagnostics Division, IL, USA). In patients with non-A non-B hepatitis, anti-HCV antibody and HCV-RNA were assessed serologically (INNOTEST^®^ HCV Ab IV, Innogenetics, Ghent, Belgium) and using polymerase chain reaction (PCR) (nested reverse transcriptase PCR by in house assay using 5′ UTR primers) [14] testing, respectively. In patients with positive HCV antibodies and RNA, exacerbation of chronic hepatitis C by other infectious agents was ruled out using reverse transcriptase PCR for HEV-RNA (in house assay using ORF1 and ORF2 primers) and serological testing [anti Epstein-Barr virus (EBV) IgM (ETI-EBV-M reverse P001605, Dia Sorin, Vercelle, Italy), anti-cytomegalovirus (CMV) IgM (AXSYM^®^ system-CMV-IgM, Abbott Laboratories, Wiesbaden, Delknheim, Germany), and anti-Toxoplasma IgM (AXSYM^®^ system-Toxo-IgM, Abbott Laboratories, Wiesbaden, Delknheim, Germany)].

Family controls were anti-HCV antibody and HCV-RNA tested using the same techniques.

### Statistical analysis

Univariate ORs and 95% confidence intervals (95% CI) were estimated for each potential risk factor using a conditional logistic regression model (to account for the matched design) and significance was assessed by the Wald test. Interactions were tested by introducing interaction terms in the model.

Since both control groups had the same odds of exposures for most exposures, as can be seen in Tables 1 to [Table pone-0007193-t002] 3, we believe that they were properly sampling the same source population of cases, i.e., those who would have gone for care at the fever hospitals had they developed acute hepatitis C. As a result, we decided to group them to increase the study power.

**Table 1:** Socio-demographic characteristics of acute hepatitis C cases and controls sex- and ±1 year age-matched, Greater Cairo, 2002-7.

	HCV cases	HAV Controls	Family Controls	OR^*^ (95% CI)	p-value
**N**	94	94	94		
**Mean age (years)**	25.9 (±5.9)	25.4 (±6.2)	25.3 (±6.0)	**NA**	**NA**
**Males**	66 (70)	66 (70)	66 (70)	**NA**	**NA**
**Education**					**<0.001**
Ability to read and write	53 (56.4)	77 (81.9)	80 (85.1)	**1**	
Illiteracy	41 (43.6)	17 (18.1)	14 (14.9)	**4.5 (2.4–8.6)**	
**Marriage duration**					**0.01**
Single	46 (48.9)	63 (67.0)	54 (57.5)	1	
Less than one year	13 (13.8)	2 (2.1)	9 (9.6)	**3.4 (1.4–8.5)**	
One year or longer	35 (37.2)	28 (29.8)	31 (32.9)	**2.2 (1.1–4.8)**	

**NOTE**: Data are no. (%) of participants. OR, unadjusted odds ratio comparing cases to the two control groups combined; CI, confidence interval. NA = non applicable because of matching.

**Table 2 pone-0007193-t002:** Health care related risk factors among acute hepatitis C cases and controls sex- and ±1 year age-matched, Greater Cairo, 2002-7.

	HCV Cases	HAV Controls	Family Controls	OR^*^(95% CI)	p-value
	N = 94	N = 94	N = 94		
***Invasive medical procedures***					
Hospital Admission	15 (16.0)	3 (3.2)	5 (5.3)	**3.8 (1.6–8.8)**	**0.002**
Admission to Intensive care	0	0	0	**–**	**–**
Surgery	8 (8.5)	2 (2.1)	1 (1.1)	**5.3 (1.4–20.1)**	**0.01**
Stitches	21 (22.3)	6 (6.4)	3 (3.2)	**5.1 (2.2–11.5)**	**<0.001**
Intravenous injections	9 (9.6)	1 (1.1)	3 (3.2)	**4.3 (1.3–14)**	**0.01**
Intramuscular injections	13 (13.8)	12 (12.8)	18(19.1)	0.8 (0.4–1.7)	0.63
Intravenous cannula	13 (13.8)	5 (5.3)	4 (4.3)	**3.3 (1.3–8.5)**	**0.01**
Catheter	0	1 (1.1)	0	**–**	**–**
Endoscopy	1 (1.1)	0	0	**–**	**–**
Laparoscopy	0	1 (1.1)	0	**–**	**–**
Abscess incision	3 (3.2)	2 (2.1)	0	3.0 (0.5–17.9)	0.22
Ascites tapping	1 (1.1)	0	0	**–**	**–**
Blood donation	5 (5.3)	5 (5.3)	3 (3.2)	1.3 (0.4–3.8)	0.70
Blood transfusion	0	0	0	**–**	**–**
***Obstetric exposures*** a					
Birth delivery	4 (14.3)	1 (3.6)	1 (3.6)	6.6 (0.7–60.9)	**0.06**
Cesarean section	3 (10.7)	0	0	**–**	**0.05**
***Dental treatment***					
Any	16 (17.0)	17 (18.1)	14(14.9)	1.0 (0.5–1.9)	1.00
Teeth extraction	12 (12.8)	11 (11.7)	5 (5.3)	1.5 (0.7–3.4)	0.30
Gum treatment	3 (3.2)	3 (3.2)	4 (4.3)	0.8 (0.2–3.2)	0.73
Filling cavities	2 (2.1)	1 (1.1)	2 (2.1)	1.2 (0.2–7.0)	0.88
Injected anesthesia	13 (13.8)	12 (12.8)	7 (7.4)	1.3 (0.6–2.8)	0.44

**NOTE**: Data are no. (%) of participants.

OR^*^, unadjusted odds ratio comparing cases to the two control groups combined CI, confidence interval.

a Only women included (N = 84).

**Table 3:** Community exposures and high risk habits among acute hepatitis C cases and controls sex- and ±1 year age-matched, Greater Cairo, 2002-7.

	HCV Cases	HAV Controls	Family Controls	OR^*^(95% CI)	p-value
***Community exposures*** c					
**Shaving at barber** d	54 (81.8)	42 (63.6)	46 (69.7)	**2.1 (0.9–4.5)**	**0.06**
Sharing razor blades^d^	4 (6.1)	7 (10.6)	11 (16.7)	0.4 (0.2–1.3)	0.14
Manicure	1 (1.1)	3 (3.2)	2 (2.1)	0.4 (0.1–3.4)	0.36
Sharing nail cutter	67 (71.3)	64 (68.1)	78 (82.9)	0.7 (0.4–1.2)	0.20
***High risk habits (males)*** d					
**Drinking alcohol**	25 (37.9)	15 (22.7)	11 (16.7)	**2.6 (1.3–5.3)**	**0.006**
**Multiple sexual partners**	8 (12.1)	2 (3.0)	5 (7.6)	**3.5 (1.1–11.6)**	**0.004**
**Illicit drug use:**					**<0.001**
No	33 (50.0)	46 (69.7)	48 (72.7)	1	
Sniffing exclusively	19 (28.8)	10 (15.2)	8 (12.1)	**3.6 (1.5–8.4)**	
IDU	9 (13.6)	2 (3.0)	1 (1.5)	**8.5 (2.1–33.8)**	
Missing	5 (7.6)	8 (12.1)	9 (13.7)	**-**	

**NOTE**: Data are no. (%) of participants.

OR^*^, unadjusted odds ratio comparing cases to the two control groups combined; CI, confidence interval.

c Neither cases nor controls were circumcised; none had ear piercing, tattoos or acupuncture.

d Since women were not asked, they were excluded from the analysis (N = 198; 66 participants per serie).

Variables with p-values<0.20 were entered into a multivariate conditional logistic regression model to simultaneously examine their independent effect. The final model was obtained through stepwise deletion of variables until all predictors left had p-values<0.05.

With this sample size, the study had an 80% power to document odds ratios (OR) above 2.0 for exposures present among 50% of controls, and 3.5 for exposures present among 5% of controls (α = 0.05; two-sided tests).

Data were analyzed using STATA 9.0 software (Stata Corporation, College Station, USA).

## Results

### Description of the study population

Ninety four cases and their 188 controls, 94 HAV controls and 94 family controls, were enrolled in the study. Mean age (±SD) of the cases was 25.9 (±5.9) years and 70% (n = 66) were males.

Case patients being symptomatic by definition, the prevalence of symptoms was the following: fever (44.7%), jaundice (95.7%), light clay stools (87.2%), dark urine (96.8%), abdominal pain (82.9%) and vomiting (63.8%). The median bilirubin level (mg/dL) was 7.2 (Interquartile range (IQR) = 4.7–11) and the median ALT level (IU/L) was 884 (IQR = 625-1350). As expected due to matching design, mean age and male proportion were similar between cases and controls (Table 1). Prevalences of exposure to studied risk factors were also similar between the two control groups, as shown in Tables 1 to [Table pone-0007193-t002] 3.

### Matched analysis

In univariate analysis, iatrogenic factors (Table 2) statistically associated with HCV infection were history of hospitalization, having major surgery, receiving wound stitches in emergency rooms, intravenous (IV) cannula, and IV injections but not intramuscular injections. In women, child birth and caesarean section were associated with increased risk of HCV infection, although the association was marginally significant for delivery (p = 0.06).

Dental treatment, even if quite common (16.5% of the control population), was not associated with increased risk of infection. This remained true after studying various dental procedures (tooth extraction, gum treatment, cavity filling and dental anaesthesia) separately.

Also in univariate analysis, illiteracy and marriage were associated with HCV transmission (Table 3). None of the community-acquired exposures showed a significant increase in HCV risk but all of studied high risk habits were associated with increases in HCV risk: drinking alcohol (OR = 2.6; 95% CI = 1.3–5.3), multiple sexual partners (OR = 3.5; 95% CI = 1.1–11.6) and IDU (OR = 8.5; 95% CI = 2.1–33.8) as well as sniffing drugs (OR = 3.6; 95% CI = 1.5–8.4).

Among the factors that could not be evaluated for their significance due to low exposure prevalence were hospitalization in intensive care unit, IV catheter, endoscope, laparoscopy, ascites tapping, biopsy, schlerotherapy for varicose, electromyogram and blood transfusion.

In multivariate analysis (Table 4), the following risk factors were independently associated with an increase in HCV risk: therapeutic intravenous injections (OR = 5.0; 95% CI = 1.2–20.2), medical stitches (OR = 4.2; 95% CI = 1.6–11.3), sniffing illicit drugs (OR = 4.4; 95% CI = 1.6–12.1) and injection drug use (OR = 7.9; 95% CI = 1.4–43.5). Being recently married versus being single (OR = 3.3; 95% CI = 1.1–9.9), and being illiterate (OR = 3.9; 95% CI = 1.8–8.5) were also associated with HCV transmission.

**Table 4:** Multivariate analysis showing factors independently associated with acute hepatitis C, Greater Cairo, 2002-7.

	Adjusted OR (95% CI) N = 275^e^	p-value
**Intravenous injections**	**5.0 (1.2–20.2)**	**0.020**
**Medical stitches**	**4.2 (1.6–11.3)**	**0.004**
Illicit drug use:		
Never	1	
**Sniffing**	**4.4 (1.6–12.1)**	**0.005**
**Injection drug use**	**7.9 (1.4–43.5)**	**0.018**
Marriage duration		
Single	1	
**Less than one year**	**3.3 (1.1–9.9)**	**0.029**
One year or longer	1.8 (0.8–4.1)	
**Illiteracy**	**3.9 (1.8**–**8.5)**	**0.001**

**NOTE**: Adjusted OR, odds ratio adjusted by all other covariates; CI, confidence interval.

e 7 participants with missing data: 6 controls with missing information on intravenous injection and one on duration of marriage.

## Discussion

In this study, three risk factors reflecting direct mechanisms of HCV transmission have been associated with HCV infection: illicit drug use, unsafe therapeutic injections and wound stitches. In addition, illiteracy and recent marriage, indirect mechanisms of transmission, were also independently associated with HCV infection. A key strength of this study is the recruitment of incident cases, allowing detailed assessment of exposures in a well-defined time period, the one to six months prior to onset of symptoms. Many published cross-sectional studies have compared individuals with and without anti-HCV antibodies to identify risk factors for infection [10], [15], [16]. These studies, although based on large numbers, suffered from drawbacks such as approximate exposure assessment over long periods of time, and the possibility that exposures followed, rather than antedated, HCV infection. Case-control studies offer a better design but still information and selection bias may happen. Regarding information bias, questionnaires in this study were administered at the time patients presented to the hospital, and prior to knowing whether they had acute hepatitis C or acute hepatitis A. Therefore, chances that the interviewer would be biased in recording exposure information for these two groups were minimised. Regarding selection bias is expected to be low since both series of controls were chosen so that they were “likely to have consulted at the study hospitals had they developed the disease of interest”, and both gave very similar prevalence estimates for all the exposures under study. Finally, our strict (±1 year) age- and gender matching maximised study power when it came to control for important potential confounders such as age and gender.

Acute infection with HCV leads to symptomatic hepatitis only in ∼15% of patients and it is most often diagnosed in the setting of post-exposure surveillance or seroconversion in high risk individuals (e.g., healthcare professionals or injecting drug users) [17]. One may wonder whether focusing on symptomatic cases might have biased the study towards the identification of risk factors more prone to lead to symptomatic forms of infection. However, we have not found in the literature evidence of factors associated with symptomatic forms of infection, except for older age [18].

Of interest, partial genotyping information is available for some patients with acute hepatitis C recruited in the same hospitals: 11 of 12 patients who underwent treatment after 4–6 months of persistent infection were infected with HCV genotype 4 [19]. And 10 out of 12 of our acute HCV participants involved in a study of HCV intrafamilial transmission were infected with genotype 4 (Paez Jimenez, personal communication). Others were infected with genotype 1. These findings are in line with the known predominance of genotype 4 among HCV-infected Egyptian subjects [20].

This study supported the important role played by invasive health care procedures in HCV transmission in this population. Intravenous injections in the past six months were associated with a five-fold increase in HCV risk in multivariate analysis. Conversely, intramuscular injections, although very common (16.0% of the control population), were not associated with any increase in HCV risk, suggesting that large hollow-bore needles, and intravenous placement of needles, are important contributors to the efficiency of HCV transmission [21]. Similarly, stitches, which involve repeated percutaneous effractions of the skin with the same needle, proved to be efficient in transmitting HCV. Other invasive medical procedures, such as endoscope, laparoscopy, or blood transfusions, were not associated with increased risk of HCV transmission, largely as a result of very low numbers of exposed individuals, limiting the possibility of estimating risks with sufficient precision. While this does not preclude increased risk of transmission associated with each procedure, it does suggest that these procedures contribute little to the HCV spread in this population, considering the very limited number of exposed individuals. Of interest, dental care, previously associated with HCV spread in Southern Italy [22] and although commonly reported, was not associated with increased risk of HCV transmission in our study. Neither were community exposures such as shaving at barbershop, circumcision, ear-piercing, tattooing, manicure or pedicure previously reported at risk in other regions [23]–[25], [26].

In high prevalence areas, contaminated medical equipment seems to be a common HCV thread [4]. Injecting drug use, leading HCV mode of transmission in low prevalence countries, has emerged in our study as a HCV risk factor in Greater Cairo. In wealthy regions [27] but also in Thailand [28], China [29], Iran [30] or Afghanistan [31], IDUs are suffering an HCV hyperendemia with HCV prevalence rates of 48–96%, due to the collective use of injecting equipment (and drug solution) and the turnover in injection partners [32] with a higher risk of acquiring HCV shortly after onset of injection (after 5 years, 50–90% of users have been exposed to HCV) [33]. In our study, intranasal drug users were also at increased risk of acquiring HCV. Sniffing has been previously identified as a HCV risk factor [34], [35] and HCV has been detected in nasal secretions [36], [37] but evidence is not consistent in all studies [38]. This association in our results could be due to IDUs reporting sniffing instead injecting practices. In any case, the high proportion of illicit drug use (11% of male controls, since women were not asked) is most probably a particularity of urban Egypt compared to rural Egypt. Scale-up of harm reduction interventions is urgently needed in Greater Cairo. This will serve not only the control of the HCV epidemic, but also that of other feared blood-borne agents such as HBV or the human immunodeficiency virus (HIV).

More puzzling is the association found between marriage and HCV infection, and particularly recent marriage. Previous studies in Egypt and elsewhere in the world gave contradictory findings regarding the risk of HCV transmission between spouses. In rural areas of Egypt, Magder et al. estimated through mathematical modelling that 6% of infected individuals acquired infection from their spouses [39]. On the other hand, cohort studies in Italy and Turkey found very low rates of intra-spousal transmission of HCV over several years of follow-up per couple [40], [41]. In this study, the risk of HCV transmission was higher during the first year of marriage, and decreased afterwards. One way to reconcile the above-mentioned findings would be to consider that upon first encounters with an HCV-infected spouse, the most susceptible individuals would become infected, while others would remain little susceptible to infection for the rest of their lives. Cohort studies, which exclude couples with both spouses already infected at baseline, would only follow less susceptible individuals, hence documenting very low rates of transmission. Unfortunately, the study was not able to identify the modes of transmission (e.g., sexual, exchange of grooming items) that may have contributed to inter-spouse transmission, hence limiting the scope of prevention messages that can be formulated at this stage.

The HCV increased risk related with illiteracy is worth mentioning. Although previously reported [42]–[44], one may have argued that this association was the result of using an improper control group with hepatitis A patients. Indeed, developing hepatitis A as an adolescent or young adult, as for controls recruited in this study, rather than during early childhood, is associated with higher standards of hygiene, and presumably higher socio-economical status. Hence, the hepatitis A control group might have been “over educated” and the acute hepatitis C group then more likely to belong to lower educational status. However, comparison with a matched group issued from relatives of hepatitis C patients also revealed the same difference in illiteracy, suggesting that low education is genuinely associated with increased risk of HCV infection. Since the OR associated with illiteracy was little modified by control for at risk exposures in multivariate analysis, one may assume that important risk practices associated with low education were not measured in this study. Then, illiteracy may reflect an increased vulnerability due to inadequacy of interpersonal networks providing support [42], [43], [45]. In this sense, it speaks for health education campaigns to be target in priority to the most deprived populations.

To conclude, these findings add to the evidence for the need to reinforce policies on HCV risk reduction in health care settings and, for the first time, suggest illicit drug use as an important HCV risk factor in urban Egypt. Effective HCV prevention programming should focus on availability of supplies, safe equipment and contaminated waste disposal in addition to adherence to universal precautions in all health care settings. Campaigns to discourage people from illicit drug consumption and harm-reduction measures such as access to sterile injection equipment or drug dependence treatment are also necessary. With increasingly effective anti-HCV therapy, coordinated mass media campaigns segmented by audience to raise awareness, promote public debate and reduce stigma towards HCV infected persons should be considered.
